# Supplementary material for: Shoe feature recommendations for different running levels: A Delphi study
Source: PLoS One. 2020 Jul 16;15(7):e0236047. doi: 10.1371/journal.pone.0236047 (PMC7365446; doi:10.1371/journal.pone.0236047)
Supplement: S1 Appendix — (DOCX) [file pone.0236047.s001.docx]

# S1 Appendix A: Footwear Features

### Crash pad

Description: Material insert in the lateral part of the heel that can be achieved by 1) incorporating a pad into the midsole construction with a softer material than the midsole, or 2) by incorporating compressible elements into the outsole of the lateral shoe heel.

Assessment: n.a.

Property categories: Yes, No, I don’t know (1,2)

### Forefoot flares

Description: A protrusion of the front section of the shoe midsole and outsole beyond the upper. Of interest are whether forefoot flares should be used and the position of the flare.

Assessment: Angle of flare to vertical. A positive flare angle indicates that the shoe midsole and outsole extend beyond the upper (Fig. S1). A negative flare angle indicates that the upper extends beyond the midsole and outsole.

Property categories: No flare, positive flare (lateral forefoot), positive flare (medial forefoot), negative flare (lateral forefoot), negative flare (medial forefoot), I don’t know


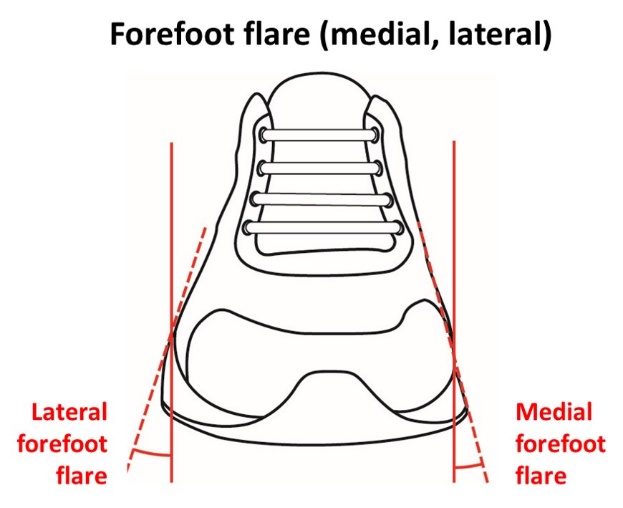


Figure S1. Illustration of the forefoot flare that was used in this Delphi study.

### Forefoot longitudinal bending stiffness

Description: The bending stiffness of the shoe (including upper) in the forefoot area around a medio-lateral axis of the shoe. Stiffness may be modulated by incorporating higher density or thicker EVA midsoles, TPU inserts, carbon fiber plates, or flex grooves.

Assessment: Currently no standard assessment methods. Possible assessments include 1) three-point bending tests (e.g., (3)), 2) flexion tests (e.g., (4)), 3) manual bending tests. In a manual bending test, a low bending stiffness corresponds to a shoe where the forefoot can easily be bent by 180-360 degrees. A high bending stiffness corresponds to a shoe where the forefoot cannot be easily bent by more than 45 degrees. A medium stiffness is in between a low and high stiffness (5).

Property categories: Low, Medium, High, I don’t know (5)

### Forefoot midsole hardness

Description: The hardness of the shoe midsole in the forefoot region.

Assessment: This feature is usually assessed with a manual durometer (Asker-C)

Property categories: Asker-C < 35, 35-40, 40-45, 45-50, 50-55, 55-60, I don’t know (6,7)

### Heel counter

Description: A reinforcement of the posterior aspect of the shoe upper surrounding the posterior heel.

Assessment: n.a.

Property categories: No heel counter, semi-rigid (reinforced with TPU / synthetic leather), rigid (reinforced with plastic element), I don’t know (8,9)

### Heel flare

Description: A protrusion of the back section of the shoe beyond the upper. Of interest are whether heel flares should be used and the position of the flare.

Assessment: Angle of flare to vertical. A positive flare angle indicates that the shoe midsole and outsole extend beyond the upper (Fig. S2). A negative flare angle indicates that the upper extends beyond the midsole and outsole.

Property categories: No flare, positive flare (lateral heel), positive flare (medial heel), positive flare (posterior heel), negative flare (lateral heel), negative flare (medial heel), negative flare (posterior heel), I don’t know (10)


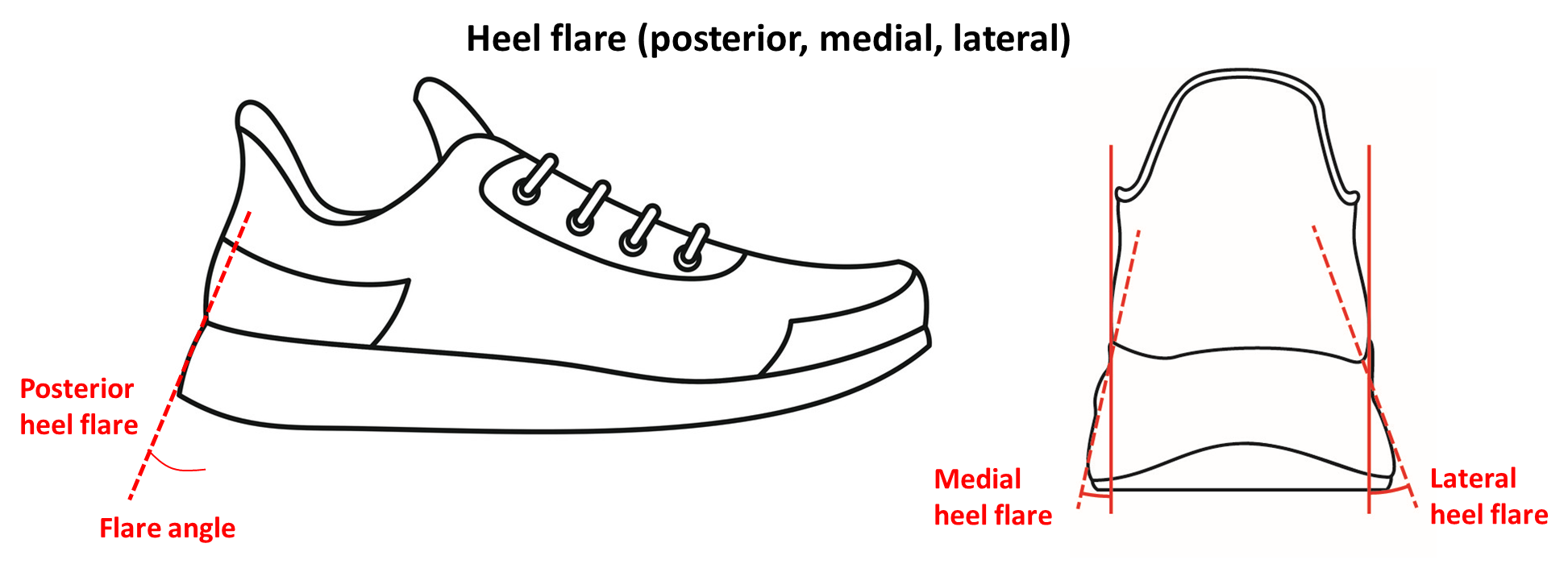


Figure S2. Illustration of the heel flare that was used in this Delphi study.

### Heel (stack) height

Description: The combined height of the outsole, midsole, potential crash-pad, and insole in the central heel region

Assessment: Height measurement.

Property categories: <14 mm, 14-32 mm, > 32 mm, I don’t know (5)

### Heel-to-toe drop

Description: The difference between the stack (heel) height and the forefoot height

Assessment: Height measurements.

Property categories: 0-4 mm, 4-8 mm, 8-12 mm, > 12 mm, I don’t know (5,11)

### Insole shape

Description: The shape of the insole (sock liner).

Assessment: n.a.

Property categories: Flat (F), arch support (AS), rounded heel (stabilizer) (RH), metatarsal pad (MP), I don’t know

### Medial post

Description: A post that is incorporated into the medial part of the shoe, either by incorporating a higher density element into the midsole (dual-density midsole) and/or by adding plastic reinforcement to the sole construction. Of interest are whether or not such elements should be used, and if yes, the position of the medial post.

Assessment: n.a.

Property categories: No, Yes (Rearfoot, RF), Yes (Midfoot, MF), Yes (Forefoot, FF), I don’t know (12)

### Midfoot longitudinal bending stiffness

Description: The bending stiffness of the shoe (including upper) in the midfoot area around a medio-lateral

axis of the shoe. Stiffness may be modulated by incorporating higher density or thicker EVA midsoles, TPU inserts, carbon fiber plates, or flex grooves.

Assessment: Currently no standard assessment methods. Possible assessments include 1) three-point bending tests (3), 2) flexion tests (4), 3) manual bending tests. In a manual bending test, a low bending stiffness corresponds to a shoe where the shoe midfoot can easily be bent by 180-360 degrees. A high bending stiffness corresponds to a shoe where the midfoot cannot be easily bent by more than 45 degrees. A medium stiffness is in between a low and high stiffness (5).

Property categories: Low, Medium, High, I don’t know (5)

### Midsole thickness

Description: The thickness (height) of the midsole in the heel region of the shoe.

Assessment: Height measurement.

Property categories: 1-5 mm, 5-10 mm, 10-15 mm, > 15 mm, I don’t know (13)

### Outsole traction

Description: The traction characteristics of the outsole on the running surface as determined by the hardness of the selected rubber and the grooves / tread pattern.

Assessment: Force required to translate/rotate the shoe relative to the running surface under axial loading (14).

Property categories: Low, medium, high, I don’t know (15)

### Rearfoot midsole hardness

Description: The hardness of the shoe midsole in the rearfoot region.

Assessment: This feature is usually assessed with an impact test or with a manual durometer (Asker-C) Property categories: Asker-C < 35, 35-40, 40-45, 45-50, 50-55, 55-60, I don’t know (6,16,17)

### Rocker (heel)

Description: A rounded (beveled) heel sole construction.

Assessment: Anterior-posterior apex (rolling point) of rocker as a percentage of shoe length with reference to the most posterior aspect of the shoe (Fig. S3).

Property categories: No rocker, small rocker (5%), mid rocker (10%), large rocker (15%), I don’t know (18)


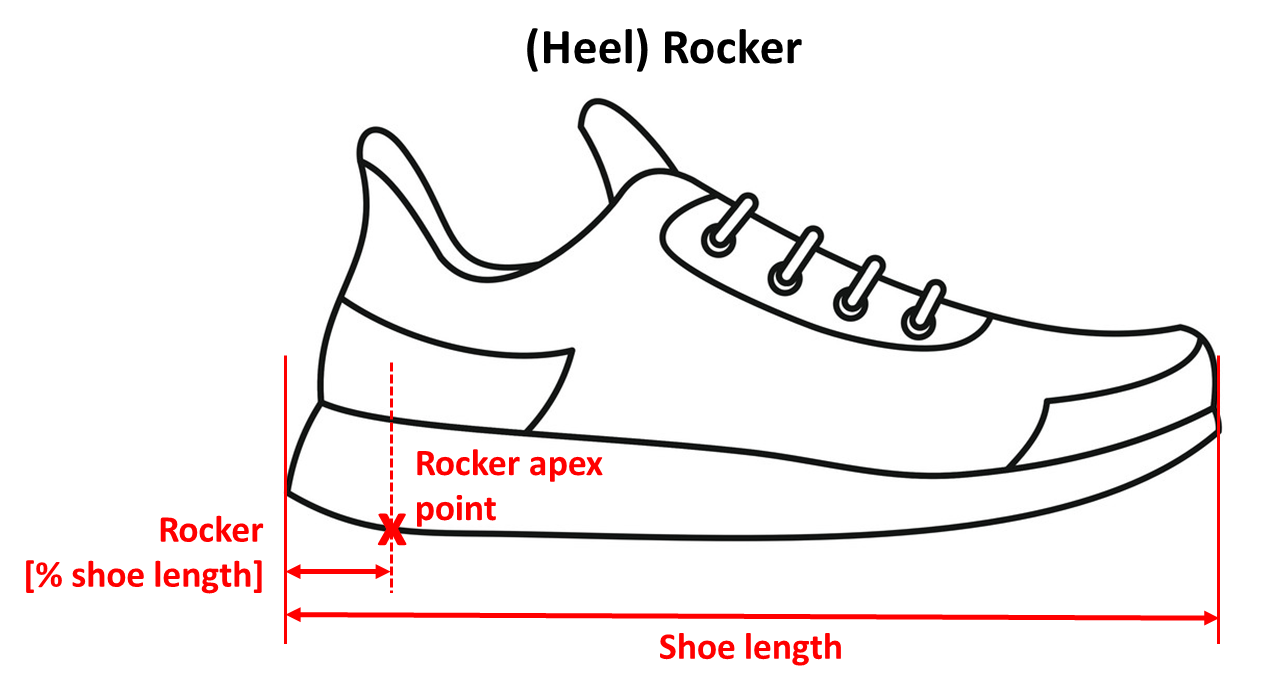


Figure S3. Illustration of the heel rocker that was used in this Delphi study.

### Shoe mass

Description: The physical mass of one shoe.

Assessment: Weight measurement.

Property categories: <175 g, 175-225 g, 225-275 g, 275-325 g, > 325 g, I don’t know (5)

### Toe spring (forefoot rocker)

Description: An upward curve of the shoe’s toe area with the apex point of the curve at the metatarsophalangeal joint.

Assessment: The angle between the horizontal and the imaginary line connecting the toe spring apex point and the shoe tip (Fig. S4).

Property categories: No toe spring, small (1-15 deg), mid (16-30), large (> 30), I don’t know (19,20)


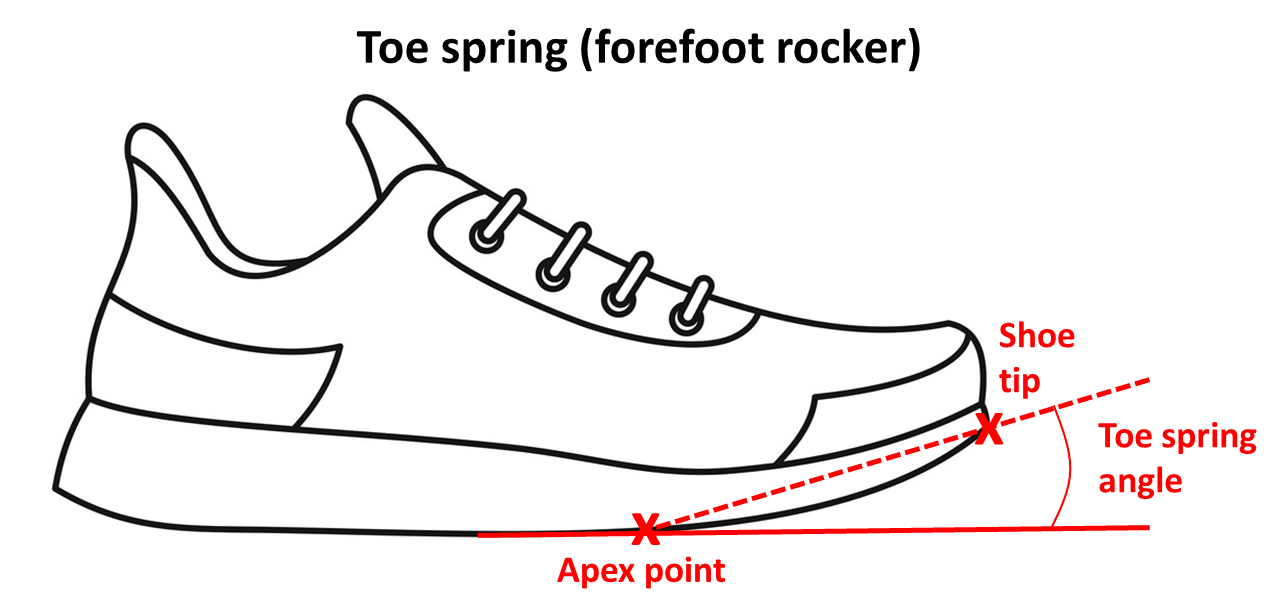


Figure S4. Illustration of the toe spring that was used in this Delphi study.

### Torsional bending stiffness

Description: The bending stiffness of the entire shoe (including upper) when rear and front part

of the shoe are rotated with respect to each other around the long axis of the shoe.

Assessment: Currently no standard assessment methods. A low torsional stiffness corresponds to a shoe where rear and front part can easily be rotated by 180-360 degrees with respect to each other. A high torsional stiffness corresponds to a shoe where rear and front part cannot be easily rotated by more than 45 degrees. A medium stiffness is in between a low and high stiffness (5).

Property categories: Low, Medium, High, I don’t know (5)

### Upper material (breathability)

Description: The ability of the upper fabric to allow moisture vapor to be transferred from the inside to the outside of the shoe.

Assessment: Currently no standard assessment methods.

Property categories: Low, Medium, High, I don’t know

### Upper material (elasticity)

Description: The elasticity of the upper, which can be modified by 1) using upper materials of different properties and 2) adding pieces of synthetic material with low elasticity for reinforcement of the toe, midfoot, or heel region (i.e. upper overlays).

Assessment: n.a.

Property categories: Low, intermediate (mid), high, I don’t know

**References**

1. Heidenfelder J, Sterzing T, Milani TL. Systematically modified crash-pad reduces impact shock in running shoes. Footwear Sci. 2010 Jun 1;2(2):85–91.

2. Sterzing T, Thomsen K, Ding R, Cheung JT-M. Running shoe crash-pad design alters shoe touchdown angles and ankle stability parameters during heel–toe running. Footwear Sci. 2015 May 4;7(2):81–93.

3. Roy J-PR, Stefanyshyn DJ. Shoe Midsole Longitudinal Bending Stiffness and Running Economy, Joint Energy, and EMG. Med Sci Sports Exerc. 2006 Mar;38(3):562–9.

4. Stefanyshyn DJ, Nigg BM. Influence of midsole bending stiffness on joint energy and jump height performance. Med Sci Sports Exerc. 2000;32(2):471–6.

5. Esculier J-F, Dubois B, Dionne CE, Leblond J, Roy J-S. A consensus definition and rating scale for minimalist shoes. J Foot Ankle Res. 2015 Aug 19;8(1):42.

6. Kersting UG, Brüggemann G-P. Midsole Material-Related Force Control During Heel–Toe Running. Res Sports Med. 2006 Jan;14(1):1–17.

7. Sterzing T, Schweiger V, Ding R, Cheung JT-M, Brauner T. Influence of rearfoot and forefoot midsole hardness on biomechanical and perception variables during heel-toe running. Footwear Sci. 2013 Jun;5(2):71–9.

8. Jørgensen U. Body load in heel-strike running: The effect of a firm heel counter. Am J Sports Med. 1990 Mar;18(2):177–81.

9. Gheluwe BV, Tielemans R, Roosen P. The Influence of Heel Counter Rigidity on Rearfoot Motion during Running. J Appl Biomech. 1995 Feb;11(1):47–67.

10. Nigg BM, Morlock M. The influence of lateral heel flare of running shoes on pronation and impact forces. Med Sci Sports Exerc. 1987 Jun;19(3):294–302.

11. Malisoux L, Chambon N, Urhausen A, Theisen D. Influence of the Heel-to-Toe Drop of Standard Cushioned Running Shoes on Injury Risk in Leisure-Time Runners: A Randomized Controlled Trial With 6-Month Follow-up. Am J Sports Med. 2016 Nov 1;44(11):2933–40.

12. Oriwol D, Sterzing T, Milani TL. The position of medial dual density midsole elements in running shoes does not influence biomechanical variables. Footwear Sci. 2011 Jun 1;3(2):107–16.

13. Law MHC, Choi EMF, Law SHY, Chan SSC, Wong SMS, Ching ECK, et al. Effects of footwear midsole thickness on running biomechanics. J Sports Sci. 2018 May 3;37(9):1004–10.

14. Schrier NM, Wannop JW, Lewinson RT, Worobets J, Stefanyshyn DJ. Shoe traction and surface compliance affect performance of soccer-related movements. Footwear Sci. 2014 May 4;6(2):69–80.

15. Worobets JT, Panizzolo F, Hung S, Wannop JW, Stefanyshyn DJ. Increasing Running Shoe Traction can Enhance Performance. Res J Text Appar. 2014 May;18(2):17–22.

16. De Wit B, De Clercq D, Lenoir M. The Effect of Varying Midsole Hardness on impact Forces and Foot Motion during Foot Contact in Running. J Appl Biomech. 1995 Nov;11(4):395–406.

17. Nigg BM, Baltich J, Maurer C, Federolf P. Shoe midsole hardness, sex and age effects on lower extremity kinematics during running. J Biomech. 2012 Jun 1;45(9):1692–7.

18. Willwacher S, Potthast W, Konrad M, Brüggemann G-P. Effect of Heel Construction on Muscular Control Potential of the Ankle Joint in Running. J Appl Biomech. 2013 Dec;29(6):740–8.

19. Trama R, Blache Y, Hautier C. Effect of rocker shoes and running speed on lower limb mechanics and soft tissue vibrations. J Biomech. 2019 Jan 3;82:171–7.

20. Long JT, Klein JP, Sirota NM, Wertsch JJ, Janisse D, Harris GF. Biomechanics of the double rocker sole shoe: Gait kinematics and kinetics. J Biomech. 2007 Jan 1;40(13):2882–90.
