# Supplementary material for: Shoe feature recommendations for different running levels: A Delphi study
Source: PLoS One. 2020 Jul 16;15(7):e0236047. doi: 10.1371/journal.pone.0236047 (PMC7365446; doi:10.1371/journal.pone.0236047)
Supplement: Attachment — Submitted filename: Response-to-Reviewers2_final.docx [file pone.0236047.s004.docx]

>> We want to thank both of the reviewers for their additional time in reviewing our manuscript. We want to let the reviewers know that we have added in the discussion figure that was removed after the first-round of reviews. We have added this figure again as it provides a more compelling case as to which important footwear features should be researched in the future. This figure utilizes data, with permission, from Hoitz et al., 2020 (recently published: [link](https://www.tandfonline.com/doi/full/10.1080/19424280.2020.1773936)).

Reviewer #1: Thank you for addressing all reviewer comments. I have no other reviewer editorial suggestions for this paper.

Overall Good work on this paper. It is interesting and pertinent.

>>Thank you for you compliments.

Reviewer #2: While many (if not most) of my original comments were addressed by the authors, several small problems still exist in my opinion. Similar to my previous assessment, these minor points negatively influence the readability and overall impact of this important work. I believe that careful attention to these points listed below will improve the manuscript.

>>Thank you for your additional comments, which have improved our manuscript. Similar and relevant comments have been grouped so that they can be addressed together.

Ensure that figures and tables (specifically table footnotes and legends) are properly formatted.

>> We have updated the figures and tables according to PLOS standards, including font type used in figures, separating the figure captions and legends, placing the table captions below the tables, and succinctly describing the figures/tables.

Table 2 - The title is too long and complicated. An appropriate title should be short and briefly describe the global goal(s) of the table. Any additional or necessary information should be described as a footnote. Additionally a footnote should be added to reflect the transition of 1 participant from one group to another.

>> We have updated the title of Table 2 to the following: “*Number of participants and their experience investigating/designing footwear.*”

>> We have updated the caption of Table 2 to the following: “*Note that one academic moved to industry between the first and second rounds of this study.*”

Tables 4 & 5 - The titles are too long and complicated. An appropriate title should be short and briefly describe the global goal(s) of each table. Any additional or necessary information should be described in footnotes. Both tables also inconsistently use the term "participants" and "respondents" to describe the expert panel. This is confusing and should be consistently reported one way or the other. Tables 4 & 5 also cover very similar material; however, their presentation is very different from one another. Consider combining or revising these tables further to enhance readability.

>>We have updated “respondents” to “participants” in Table 4.

>>We have updated the title of table 4 to: “*Percent of participants that agreed upon the importance of shoe features.*”

>>We have updated the title of table 5 to: “*Shoe feature properties that was most frequently chosen for each running level.*”

Table 5 - Putting the "*" next to the percentage seems somewhat redundant. Consider putting this next to the property running level.

>>We have removed the asterisk from Table 5.

Ensure citations are properly and consistently formatted (see 41 and 42 for examples).
>> Thank you for bringing this to attention. We have updated the citations mentioned (see below) and checked all other citations.

“41. Tilp M. Benno M. Nigg, Maurice M. Mohr & Sandro R. Nigg – New paradigms in running injury prevention. Curr Issues Sport Sci. 2019 May 7;4(100).

42. Oh K, Park S. The bending stiffness of shoes is beneficial to running energetics if it does not disturb the natural MTP joint flexion. J Biomech. 2017 Jan 18;533:127–35.”

Ensure double spacing throughout the text and eliminate unnecessary spacing.

>> We have eliminated unnecessary spacing and formatted table/figure captions to double spacing.

Line 82 - The term "footwear research experts" seems somewhat inappropriate as active researchers were not explicitly recruited and may also only make up a portion of your participants. Consider simplifying by using the term "footwear experts." Even though experts are often involved with or active in conducting research, they do not necessarily need to participate in research to be labeled as experts.

Line 96 - Again, the use of the term "footwear research experts" does not likely accurately describe the entire population of experts recruited.

Line 417 - The term "footwear research experts" is a somewhat counterintuitive term to use given that researchers make up only a portion of your participants as well as your audience. Consider simplifying by using the term "footwear experts."

>> We have updated “*footwear research experts*” to “*footwear experts*” throughout the manuscript.

Line 82 - Use of the word "powerful" seems subjective. Furthermore, this sentence seems somewhat redundant given the following line describing the successful use of a Delphi study in the field. Consider combining these 2 statements and using less conjecture.
>>We have combined the two sentences as recommended:

“*An understanding of how footwear experts make decisions about different footwear features and their properties can be obtained through gathering and summarizing opinions of experts in the field of running biomechanics and footwear using a Delphi study.*”

Lines 106 & 123 - Figure 1 should be moved to, and initially referenced in, the "Results: Participation" section.

>>We have moved Fig. 1 into the results section.

Line 113 - It is stated that individuals without at least 2 years of research experience were excluded from participation. As stated, this criteria seems to have been ignored. The definition of "research," especially within the context of a peer-reviewed journal, seems likely to exclude a majority of the persons tapped to participate, as well as a number of individuals that may have participated in the study. This point needs to be clarified or revised.

>>We have removed the word “research” and have update the sentence to:

“*Participants were excluded if they had under two years of experience related to running footwear in their respective fields of expertise*”

Line 117 - "and were able to provide feedback..." The use of the word "able" is somewhat misleading given the dual nature of the word in context. The reader could interpret that all round 1 participants continued through rounds 2 and 3; or the reader could think that all participants were invited to again participant in subsequent rounds. Additionally, if an individual did participate in say round 1 but did not in round 2, were they invited back for round 3? This broader question should be more explicitly detailed (lines 119-121 seem to vaguely describe this question).

>> We have removed the word “able” and clarified the text as to the participation with the following:

*“The footwear experts completed web-based surveys through QuestionPro (questionpro.com) and could provide feedback after the completion of each round of this Delphi study. The participants that completed the first-round survey were invited to participate in the second-round. Similarly, the participants that completed the second-round survey were invited to participate in the third round.”*

Line 188 - Tense shift "are." The question as stated does not broadly apply to each feature as I believe the authors intend. Consider: "In the first-round, participants were asked if [each] footwear features [was] important when designing..."

>> We have updated the text as recommended.

Lines 190-191 - Restructure the sentence using basic "if - then" logic to simplify reading.

Lines 191-192 - This sentence should likely come before the prior one as it establishes a standard for your selection criteria; however, additional information is also necessary to justify why 51% or 80% thresholds were not used.

>>We have updated the text to the following:

“*If over 75% [a similar threshold to (22,23)] of the first-round participants selected option (a), the footwear feature was defined as important.*”
>>We have included the following citations to support the selection of 75% threshold:

22.	Cook C, Brismée J-M, Fleming R, Sizer PS. Identifiers Suggestive of Clinical Cervical Spine Instability: A Delphi Study of Physical Therapists. Phys Ther. 2005 Sep 1;85(9):895–906.

23.	Binkley J, Finch E, Hall J, Black T, Gowland C. Diagnostic Classification of Patients with Low Back Pain: Report on a Survey of Physical Therapy Experts. Phys Ther. 1993 Mar 1;73(3):138–50.

Line 206 - "Most footwear feature properties were obtained through literature..." What literature: "the literature," "a review of the literature," etc? More information similar to the features list is necessary.

>>This information is within S1 Appendix. We have included a call-out in the sentence and reads as such:

“Most footwear feature properties were defined based on the reviewed footwear literature (see S1 Appendix);”

Line 240 - Statistical analysis was not performed. Descriptive statistics are not analytic statistics.

>> We have added the following to the statistics portion of our manuscript:

“*Paired statistical analyses were performed to determine if the running level definitions improved through the three rounds of this Delphi study. A Friedman’s test was performed utilizing the subjective ratings from the respondents that participated in all three rounds of the study (N = 24). If the Friedman’s test revealed a significant effect, follow-up Wilcoxon signed-rank tests with a Bonferroni correction were performed to investigate pairwise differences between the individual rounds. The significance level α was set to 0.05 for all statistical tests.*”

>>And the following to the results section:

*“The respondents’ rating of the running level definitions improved as the Delphi study progressed, χ2 (2, N=24) = 13.95, p=0.0009.”*

and

“*The increase in the running level scores between the first and third rounds was statistically significant (p=0.006).*”

Lines 240-241 - What version of MATLAB and Illustrator were used?

>>We have updated the text to include version numbers:

“*All analyses were performed in MATLAB (version 2019a, MathWorks, Natick, MA, USA).* *Figures were created in MATLAB and Adobe Illustrator (version 22.1, San Jose, CA, USA).*”

Lines 254-263 - This sentence is very difficult to read and covers too much material.

>>We have broken up the mentioned sentence and now reads as such:

*“The increased running level ratings were accompanied by changes to the running level definitions. The changes to the “novice” running level definition for the second-round were: increased running experience to one year and replaced “stress management” with “enjoyment” for running motivation. The changes to the “recreational” running level definition for the second-round were: increased running experience to greater than one year and replaced “stress management” with “enjoyment” for running motivation. The changes to the “high-caliber” running level definition for the second-round were: increased running habits to >4 sessions/week and >50 km/week, replaced “stress management” with “enjoyment” for running motivation, re-order the running motivation to 1) Competition, 2) Improve general heath, and 3) Enjoyment, and re-order the priorities for footwear design to 1) Improve performance, 2) Reduce injury risk, 3) Improve comfort. We also specified the running performance as males between the ages of 18 to 34.”*

Line 323 - I take pause again with the use of the word "researching" here to broadly describe your population of experts. No clear attempts to recruit ONLY those actively involved with footwear research were described by the authors. Even though experts are often involved with or in research, they do not necessarily need to be in order to be labeled as experts.

>>We have replaced “*researching*” to “*examining*”

Line 333 - Instead of the word "topics" consider using "features" to clearly identify what has been studied. Additionally, "i.e." may have been used incorrectly here. Consider revising according to your intended list of these features (if you are only listing and describing only 2 features, then a colon is more appropriate). (ALSO see the next statement).

>>We have revised the sentence as recommended.

Line 334 - This sentence should not begin with the citation ["(12)"]. Identify the author(s) according to the reference guidelines and use the numeric citation at the end. The use of the word "topics" is somewhat vague, consider "features" as that is what you are referencing. Also consider combining this sentence with the previous as they are saying the same thing.

>> We have eliminated the sentence in question and implemented the citation at the end of the topic sentence.

Lines 335-337 - This sentence seems to assume that the listed publications were described in the previous sentence(s) through the use of the word "these;" however none of these works were described or referenced in regard to the point being made. Please reorganize this sentence, and/or the previous sentences, to ensure readability.
>>We have updated the corresponding sentence to the following:

“*Previous research has shown that a softer rearfoot midsole can reduce ground reaction force loading metrics such as vertical loading rate or peak impact forces (25–27), which have been hypothesized to reduce running-related injuries (28,29).*”

Lines 423-424 - The word "research" should be in the past tense. This sentence also seems somewhat empty given that 2 features are identified earlier in the "Discussion."

>>We have updated the tense to “researched”

An additional limitation that needs to be addressed is the transferability of these results to various running surfaces.

>>We have added the following to the limitations section:

“*These results may also not be generalizable to different running surfaces/terrains as we asked participants to only consider running on a hard surface.*”
